# Contemporary clinical management of acute pulmonary embolism: the COPE study

**DOI:** 10.1007/s11739-021-02855-0

**Published:** 2022-01-04

**Authors:** Cecilia Becattini, Giancarlo Agnelli, Aldo Pietro Maggioni, Francesco Dentali, Andrea Fabbri, Iolanda Enea, Fulvio Pomero, Maria Pia Ruggieri, Andrea Di Lenarda, Michele Gulizia

**Affiliations:** 1 Internal, Vascular and Emergency Medicine-Stroke Unit, University of Perugia, Piazzale Lucio Severi 1, 06129 Perugia, Italy; 2 ANMCO Research Center of the Heart Care Foundation, Florence, Italy; 3 Department of Clinical and Experimental Medicine, Insubria University, Varese, Italy; 4 Emergency Department, “Presidio ospedaliero Morgagni-Pierantoni”, Forlì, Italy; 5 U.O.C. Medicina e Chirurgia d’Urgenza, A.O.R.N. “S. Anna e S. Sebastiano”, Caserta, Italy; 6 Division of Internal Medicine, Michele and Pietro Ferrero Hospital, Verduno, Italy; 7 U.O.C. Medicina d’Urgenza e Pronto Soccorso, AO San Giovanni Addolorata, Rome, Italy; 8 Cardiovascular Center, University Hospital and Health Services of Trieste, Trieste, Italy; 9 Division of Cardiology, Garibaldi-Nesima Hospital, Catania, Italy; 10 Heart Care Foundation, Florence, Italy

**Keywords:** Pulmonary embolism, Registry, Outcome, Anticoagulants

## Abstract

**Background:**

New management, risk stratification and treatment strategies have become available over the last years for patients with acute pulmonary embolism (PE), potentially leading to changes in clinical practice and improvement of patients’ outcome.

**Methods:**

The COntemporary management of Pulmonary Embolism (COPE) is a prospective, non-interventional, multicentre study in patients with acute PE evaluated at internal medicine, cardiology and emergency departments in Italy. The aim of the COPE study is to assess contemporary management strategies in patients with acute, symptomatic, objectively confirmed PE concerning diagnosis, risk stratification, hospitalization and treatment and to assess rates and predictors of in-hospital and 30-day mortality. The composite of death (either overall or PE-related) or clinical deterioration at 30 days from the diagnosis of PE, major bleeding occurring in hospital and up to 30 days from the diagnosis of PE and adherence to guidelines of the European Society of Cardiology (ESC) are secondary study outcomes. Participation in controlled trials on the management of acute PE is the only exclusion criteria. Expecting a 10–15%, 3% and 0.5% incidence of death for patients with high, intermediate or low-risk PE, respectively, it is estimated that 400 patients with high, 2100 patients with intermediate and 2500 with low-risk PE should be included in the study. This will allow to have about 100 deaths in study patients and will empower assessment of independent predictors of death.

**Conclusions:**

COPE will provide contemporary data on in-hospital and 30-day mortality of patients with documented PE as well as information on guidelines adherence and its impact on clinical outcomes.

**Trail registration:**

NCT number: NCT03631810.

## Introduction

Acute pulmonary embolism (PE) is a common and potentially life-threatening disease [1–4]. The incidence is estimated to be 0.5–1.5 per thousand person-years [1]. Mortality in patients with acute PE ranges from less than 1% to more than 30% during the hospital stay, depending upon the clinical presentation and comorbidities [5–7]. According to currently available evidence, international guidelines recommend to tailor diagnostic workout, patient disposition (intensive care unit vs. medical wards vs. short hospital stay or home treatment) and acute treatment (thrombolysis vs. anticoagulant treatment) to the estimated risk for short-term death [8–14].

The most recent data on the acute phase management and clinical course in large cohorts of patients with acute PE were published almost 10 years ago [5–7]. However, the clinical management and course of PE have been changing over the last years [4]. In fact, new management pathways (home treatment/early discharge), risk stratification tools and treatment strategies (direct oral anticoagulants) have become available with potential influence on patient course and outcome. For these reasons, the assessment of the contemporary clinical management of patients with acute PE would provide valuable information. In addition, assessment in different settings (cardiology, emergency and internal medicine departments) would offer a complete scenario of current management strategies in patients with acute PE.

In this paper, we report the study design of the COntemporary clinical management of patients with acute Pulmonary Embolism (COPE) study in the context of the current literature and recent guidelines.

The aim of the COPE study is to assess contemporary management strategies in patients with acute, symptomatic, objectively confirmed PE concerning diagnosis, risk stratification, hospitalization and treatment and to assess rates and predictors of in-hospital and 30-day mortality. Adherence to guidelines of the European Society of Cardiology (ESC) and its association with in-hospital and 30-day mortality will also be assessed. As this study was planned in 2016 and patient accrual started on 2018, the study protocol has been based on the guidelines of the European Society of Cardiology released in 2014 [10].

## Methods

### Study design and setting

COPE is a prospective, non-interventional, multicentre study in patients with symptomatic, objectively diagnosed acute PE (either first or recurrent episode) evaluated at Cardiology, Emergency and Internal Medicine Departments in Italy.

**Fig. 1:**
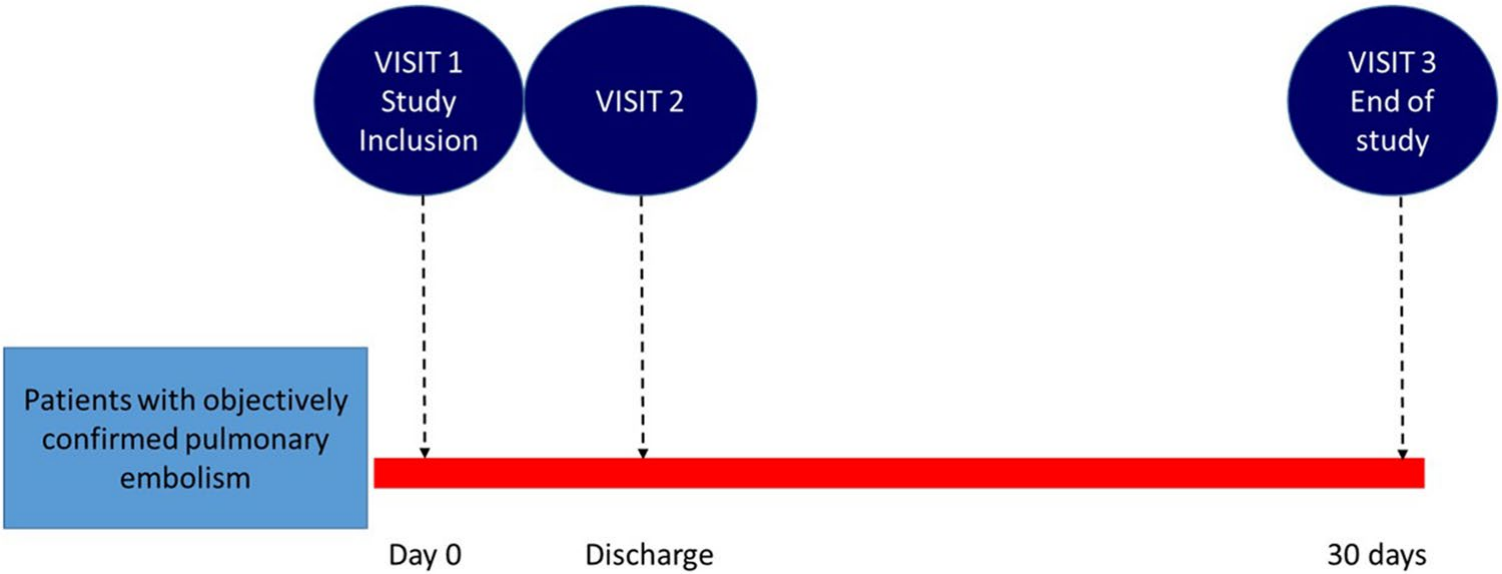
Study design

Patients are evaluated at the time of diagnosis, at discharge and at 30 days (± 4) from the index PE (Fig. 1). For patients discharged beyond 30 days from index pulmonary embolism study end -and study outcome assessment- is set at 30 days. Diagnostic workout, risk stratification and treatment strategies are at the discretion and responsibility of the attending physician. Physicians are encouraged to prescribe medications according to their usual standard of care.

COPE is a no-profit study promoted by the University of Perugia and the Fondazione per il Tuo cuore onlus-ANMCO (Associazione Nazionale Medici Cardiologi Ospedalieri), with the collaboration of SIMEU (Società Italiana Medicina Emergenza Urgenza) and FADOI (Federazione delle Associazioni dei Dirigenti Ospedalieri Internisti). The Steering Committee has the full responsibility for the study design, protocol, study oversight, data analysis, and writing and submission of the manuscript for publication. The study is supported by an unrestricted grant from Daiichi Sankyo Europe and Daiichi Sankyo Italia. The study was approved by the Ethic Committees and Institutional Review Boards of the coordinating center and at each participating center.

### Participants

Patients 18 years old or older with symptomatic objectively confirmed acute PE are included in the study after release of informed consent in accordance with the ethical standards of the responsible committee on human experimentation (institutional and national).

Criteria for diagnosis of PE are reported in Table 1. For critically ill patients unable to provide the informed consent due to very severe clinical conditions delayed consent is allowed, until more favourable clinical conditions allow them to receive the appropriate information. Patients who die before giving informed consent are also included in the study, upon authorization of local Institutional Review Board. In this case, it is strongly suggested to obtain agreement from relative(s). As this is a non-interventional study, no inclusion/exclusion criteria apply, except for exclusion of patients participating in controlled trials on the management of acute PE.

**Table 1:** Diagnosis criteria for acute pulmonary embolism by different diagnostic tools

Instrumental test	Diagnostic criterion
CT angiography	An intraluminal filling defect at computed tomography angiography
Lung scan	A perfusion defect of at least 75% of a segment with a local normal ventilation result (high probability) on ventilation/perfusion lung scan (VQ scan)
	A perfusion defect of at least 75% of a segment with a normal chest X Ray
	Intermediate probability perfusion lung scan associated with objective diagnosis of deep vein thrombosis in patients with symptoms of acute PE
Pulmonary angiography	An intraluminal filling defect, or a new sudden cut-off of vessels more than 2.5 mm in diameter at pulmonary angiogram
Lower limbs ultrasonography	A proximal deep vein thrombosis in a patient with symptoms of acute PE
Echocardiography (in patients with cardiogenic shock)	Right ventricle dysfunction

Approvals and administrative procedures were completed in first trimester 2018 and the first patient in was on April 2018. Patient accrual was completed on first trimester 2021.

### Study objectives and outcomes

Study objectives and outcomes are reported in Table 2. The co-primary study outcomes are in-hospital death and death at 30 days from the diagnosis of PE. For all patients, the cause of death is reported as assessed by the attending physician and centrally adjudicated by an independent Critical Event Committee unaware of physician classification.

**Table 2:** Study outcomes definition

Study outcomes	Study outcome definition
*Co-primary outcomes*	
In-hospital mortality	Death will be classified as due to: PE, major bleeding, cancer, cardiovascular disease not pulmonary embolism, sudden unexplained death, other non-cardiovascular death, unknown cause. Pulmonary embolism-related death is defined as: 1. Death where pulmonary embolism is the most probable cause or 2. Based on objective diagnostic testing performed before death or as assessed at autopsy (autopsy is not mandatory).
30-day mortality	
*Secondary outcomes*	
Death or clinical deterioration at 30 days	Clinical deterioration defined as occurrence of at least 1 of the following [25] 1. Need for cardiopulmonary resuscitation 2. Systolic blood pressure < 90 mm Hg for at least 15 min, or drop of systolic blood pressure by at least 40 mm Hg for at least 15 min, with signs of end-organ hypoperfusion (cold extremities, or urinary output < 30 mL/h, or mental confusion) 3. The need for catecholamine infusion (except for dopamine at a rate of < 5 μg kg^−1^ min^−1^) to maintain adequate organ perfusion and a systolic blood pressure of > 90 mm Hg
PE-related death or clinical deterioration at 30 days	
Contemporary clinical management strategies and mortality in PE patients by Admission at Cardiology, Emergency or Internal Medicine Departments Belonging to different categories of risk according to the ESC guidelines	
Adherence to current guidelines on the management of acute pulmonary embolism released by the ESC concerning diagnosis, risk stratification, hospitalization and treatment	Adherence to current guidelines will be evaluated by the following Diagnosis: time from diagnosis to initiation of anticoagulant treatment; Number of diagnostic tests applied and their sequence based on estimated pre-test probability of pulmonary embolism Prognostic assessment: type, number and timing (within 24 h) of tests performed for prognostic assessment Acute phase treatment: systemic thrombolysis or percutaneous manoeuvre associated with intravenous heparin for at least 48 h in hemodynamically unstable patients; the number, dose and sequence of antithrombotic agents according to currently validated regimens Discharge: home treatment or short hospital stay (< 48 h) in patients with low-risk pulmonary embolism
Major bleeding	Acute clinically overt bleeding associated with one or more of the following (1) decrease in hemoglobin ≥ 2 g/dl (1.2 mmol/L); (2) transfusion of ≥ 2 units of packed red blood cells; (3) bleeding that occurs in at least one critical site [intracranial, intra-spinal, intraocular (within the corpus of the eye), pericardial, intra-articular, intramuscular with compartment syndrome, or retroperitoneal]; (4) fatal bleeding; (5) bleeding that necessitates acute surgical intervention;
Clinically relevant non-major bleeding	Acute clinically overt bleeding that does not meet the criteria for major and consists of (1) any bleeding compromising hemodynamics; (2) spontaneous hematoma larger than 25 cm^2^, or 100 cm^2^ if there was a traumatic cause; (3) intramuscular hematoma documented by ultrasonography; (4) epistaxis or gingival bleeding requiring tamponade or other medical intervention or bleeding from venipuncture for > 5 min; (5) hematuria that was macroscopic and spontaneous or lasted for ≥ 24 h after invasive procedures; (6) hemoptysis, hematemesis or spontaneous rectal bleeding requiring endoscopy or other medical intervention; (7) any other bleeding with clinical consequences for a patient such as medical intervention, need for unscheduled contact with a physician, or temporary cessation of a study drug, or associated with pain or impairment of activities of daily life

The secondary study outcomes are (1) death or clinical deterioration at 30 days from the diagnosis of PE; (2) PE-related death or clinical deterioration at 30 days from the diagnosis of pulmonary embolism; (3) adherence to current guidelines on the management of acute PE released by the ESC regarding diagnosis, risk stratification and treatment and (4) major bleeding occurring in-hospital and at 30 days from the diagnosis of PE. The definition of PE-related death is reported in the Table 2.

Study outcomes will be evaluated in the overall study population with descriptive purposes and compared among patients admitted in Cardiology, Emergency or Internal Medicine Departments as well as among patients belonging to different categories of risk according to the ESC guidelines.

Adherence to ESC guidelines 2014 will be assessed concerning: diagnosis, risk stratification, treatment and global management (the composite of diagnosis, risk stratification and treatment). For the assessment of adherence, six process indicators were selected, based on recommendations from ESC guidelines [10]. The indicators are reported in Table 2.

The primary safety outcome is major bleeding according to ISTH criteria, occurring up to 30 days from the diagnosis of index PE (Table 2). Major bleeding events will be further sub-classified as life-threatening or non-life threatening. A life-threatening major bleed is defined as a bleeding event that is either intracranial or is associated with haemodynamic compromise requiring intervention.

Secondary safety outcomes are (a) major bleeding according to ISTH definition plus all bleeding that led to presentation to an acute care facility or hospitalization, occurring up to 30 days from the diagnosis of index PE (b) clinically relevant non-major bleeding defined according to ISTH criteria.

### Data sources and measurements

Scheduled assessments for the study are presented in Table 3. All data are collected from information routinely recorded in the patient files/medical records, during clinical visits or during telephone follow-up interviews. All these data are available as part of the routine treatment. All performed examinations depend on the discretion and clinical routine of the physician/site. No diagnostic or monitoring procedures and no examinations, laboratory tests or procedures are applied to the patients as part of this non-interventional study others than those performed as standard of care.

**Table 3:** Scheduled assessments during the study period

Timing	Collected data
Baseline/enrolment	Demographics, vital signs, medical history (past and current status) Current PE: diagnosis, risk stratification, treatment (agents, time of initiation, dose, duration) Concomitant cardiovascular and non-cardiovascular treatments Laboratory examinations Other VTE-relevant patient information e.g. bleeding disposition, thrombocytopenia, alcohol consumption, frailty, hospitalisation/outpatients management related to current PE
At discharge	Date of discharge Vital status and current vital signs (i.e. dead/alive, blood pressure, oxygen saturation, etc.) Clinical deterioration, bleeding during the hospital stay (i.e. date, treatment strategies) Anticoagulant treatment during the hospital stay (i.e. agent, dose, initiation/stop date) and at discharge Concomitant cardiovascular and non-cardiovascular treatments
At 30 days	Vital status and current vital signs (i.e. dead/alive, blood pressure, oxygen saturation, etc.) Clinical deterioration, bleeding events (i.e. date, treatment strategies) Anticoagulant treatment (i.e. agent, dose, initiation/stop date) Concomitant cardiovascular and non-cardiovascular treatments, including non-pharmacological treatments Follow-up examinations if available (i.e. laboratories as renal function, hemoglobin, cardiac enzymes; cardiac as electrocardiography, echocardiography, computed tomography) Hospitalisations (i.e. related to index pulmonary embolism or other causes)

### Statistical methods

For all study endpoints, the estimates of overall rates of in-hospital and 30-day events from diagnosis of PE with 95% confidence interval will be calculated.

Incidence rates of the study outcome events will be described and compared in subgroups of patients defined by Department of admission (Cardiology, Internal Medicine, Emergency Medicine) as well as by risk of death category according to the ESC guidelines (high, intermediate and low). Comparisons will be done via Cox proportional hazard regression models presenting hazard ratios and corresponding 95% confidence intervals, having patients admitted at Cardiology or low-risk patients as reference groups, respectively. If differences in baseline characteristics will be detected, these variables will be added to the model as additional covariates. Kaplan–Meier estimates will be calculated for the occurrence of death at 30 days and of death or clinical deterioration at 30 days from diagnosis of PE.

Concerning the definition of adherence to current guidelines, firstly, the number of indicators that each patient met will be counted and divided by the total number for which the patient was eligible, obtaining the proportion of adherence. The adherence score is defined as the ratio of the diagnostic tests/prognostic evaluations/treatment actually prescribed to those that should theoretically have been prescribed. Adherence will be calculated separately for each step (diagnosis/prognostic stratification/treatment) and globally for the overall patient’s management. The theoretical diagnostic/prognostic/treatment/global scores will be calculated for every patient, taking into account guideline-based eligibility criteria, contraindications to tests/drugs or treatments. The adherence score will be calculated for each patient by summing the points attributed as follows: (a) for diagnostic workup: 0 points for use of not recommended diagnostic tests according to pre-test clinical probability; 0.5 points for use of not validated sequence or 1 point for use of recommended sequence of diagnostic tests; (b) for prognostic evaluations: 0 points for no use of tests when indicated; 0.5 points for use of not recommended tests or 1 point for use of recommended tests; (c) for treatment: 0 points for non-prescription of a given treatment (i.e. thrombolysis) in the absence of contraindications, 0.5 points for use of not validated regimen or 1 point for use of validated regimen.

For each step of diagnosis, risk stratification and treatment, the score ranges from 0 (very poor) to 1 (excellent) and we define three levels of adherence: good adherence (score = 1); moderate adherence (score > 0.5 to < 1) and poor adherence (score ≤ 0.5). In this study, the term ‘adherence’ relates solely to physicians following guidelines, not to patient compliance.

We define as adherent to the care pathway a patient with a proportion of met indicators equal or greater than 80%. Sensitivity analyses will be performed at different cut-offs, and considering adherence as an ordinal and a continuous variable. A directed acyclic graph (DAG) will be constructed [14] to represent assumptions regarding the underlying associations between guideline adherence, survival and a set of clinical and socioeconomic variables. The DAG utilizes these assumptions to select the potential confounders, rather than relying simply on the statistical associations observed in the data. The selected confounders are then used in the statistical analysis aiming at minimizing the risk of a biased evaluation of the association between adherence to guidelines and survival.

### Sample size

The sample size was calculated to observe at least 100 deaths at 30 days in the study population, to have enough power to describe incidence rates of study outcome events in the overall study population and in patients at high, intermediate and low-risk of death according to ESC guidelines.

Based on the estimated prevalence of patients at high, intermediate and low-risk in a contemporary, prospective cohort of patients with acute PE and expecting an incidence of death at 30 days of 3% and 0.5% for patients with intermediate or low-risk, respectively, it is estimated that 2100 patients with intermediate PE and 2500 with low-risk PE should be included in the study. In the same time frame, it is estimated that around 400 patients with high-risk PE will be observed. The expected mortality in these patients is 10–15%. According to these estimates, we would be able to have about 100 deaths at 30 days in study patients and this will empower assessment of independent predictors of death. Besides already known predictors (hypotension, right ventricle dysfunction—imaging and BNP—and increased troponin), the role of comorbidities and clinical features as well as adherence to current guidelines on PE (risk stratification adequate, appropriateness of acute treatment) as determinants of prognosis will be assessed. The same features will be tested as predictors of death or clinical deterioration.

The inclusion of patients in each risk category (low, intermediate and high risk for death) could be stopped when the predicted number of patients is complete. In that case an alert signal will appear in the e-CRF once the estimated sample is completed.

## Discussion

The description of contemporary short-term mortality and management strategies in patients with acute PE is essential to drive future clinical research and inform health systems organization. In this context, the definition of predictors of death and the description of the association between guidelines adherence and clinical course is essential.

Acute PE is a common condition associated with substantial mortality in the short-term after diagnosis. Several international societies released evidence-based guidelines on the management of patients with acute PE [8–14]. Consensus exists that as in other cardiovascular conditions, clinical management of PE patients in the acute phase should be framed according to disease severity to reduce mortality and optimize resource allocation. In this context, the guidelines from the ESC recommend the use of a well-defined risk-driven patient management in the acute phase, including the use of clinical scores and right ventricle assessment [10, 11]. This approach is not completely endorsed by other guidelines and scientific societies, mainly due to unconvincing evidence that in hemodynamically stable patients with acute PE a risk driven approach is required to adapt clinical management. All guidelines recommend to concentrate special efforts for the management of patients with acute PE presenting in shock or hemodynamic impairment as short-term mortality in these patients can be as high as 30–50%; these patients categorized as at ‘high-risk’ of death according to the ESC Guidelines and as affected by ‘massive PE’ according to the American guidelines should rapidly proceed to a definitive diagnosis and reperfusion by thrombolytic therapy to get reduction in mortality (OR 0.53, 95% CI 0.32–0.88) [15]. The vast majority of patients with acute PE are hemodynamically stable at presentation. In this setting, pre-test clinical probability should drive the diagnostic work-up to avoid useless examinations and exposure to radiation, with acceptable failure rates and risk stratification should drive decision making on hospitalization and acute phase treatment [16–18]. However, optimal strategies for risk stratification in this context are controversial [19]. Identification of patients at low risk of death can be made by clinical models or exclusion of right ventricle dysfunction [9–11, 19, 20]. Recently, a prognostic role of right ventricle assessment has been confirmed also in PE patients at low-risk according to clinical models [21]. In patients with acute PE and low risk for death, out-patient management (hospitalization < 48 h) has shown safe in clinical studies [22–24]; however, the feasibility of out-patient management in clinical practice is debated. Hemodynamically stable patients with evidence of right ventricle dysfunction or injury should be hospitalized as the risk for death in the short term is about twofold that of patients with no signs of right ventricle overload (OR 1.94 95% CI 1.23–3.06 for echocardiography, OR 1.64 95% CI 1.06–2.52 for CT-angiography, OR 5.90 95% CI 2.68–12.95 for increased troponin) [10].

For all patients with acute PE, regardless of risk category for short-term death, anticoagulants are the mainstay for the treatment as these drugs reduce recurrent venous thromboembolism by more than 90% [8–14]. Direct oral anticoagulants (DOACs) used in fixed doses with no need for monitoring are the new standard for the treatment of venous thromboembolism [25–30]. It remains to be addressed whether DOACs can be an appropriate treatment for the overall severity spectrum of patients with acute PE. The real-life adherence to current guidelines on the management of patients with acute PE concerning diagnosis, risk stratification and treatment (including physicians’ preferences on anticoagulation therapies) and whether adherence may influence clinical course is unknown. COPE will provide contemporary evidences on clinical course and guidelines adherence of patients with acute PE in clinical practice.

COPE is a prospective cohort study. The non-interventional design of the study, the potential to include patients in critical conditions or dead and the limited number of exclusion criteria facilitate consecutive enrolment. Randomized clinical trials are essential to assess the role of diagnostic and therapeutic interventions as well as management strategies in a specific clinical setting. The results of these studies are the basis to drive clinical practice guidelines. However, randomized clinical trials generally conducted in selected specialized centers, might not enroll patients who are as heterogenous as those seen in clinical practice. Older and frail patients with contraindications to anticoagulant treatment, patients with severe clinical presentation and multimorbidity continue to be underrepresented in contemporary clinical trials on treatment approaches for patients with acute PE. Registries and other observational data may serve as valuable tools for understanding the level of adherence and the effects of guideline-recommended management in heterogeneous patient populations and settings of care. Central authorities have recently renewed the importance of patient registry studies to collect uniform data on a population defined by a particular disease, condition, or exposure, and that is followed over time [Patient Registry Initiative-Strategy and Mandate of the Cross-Committee Task Force (europa.eu)].

In the COPE study, the participation of Cardiology, Emergency and Internal Medicine Departments allows the inclusion of the whole severity spectrum of acute PE and the evaluation of differences in clinical management among different clinical settings and among academic vs. non-academic hospitals.

For this purpose, study centers were selected to guarantee representativeness for their medical activities as much as possible, and for their interest in the study.

Several large cohort studies and registries have been completed or are currently ongoing in patients with acute venous thromboembolism. All these are prospective, international, cohort studies and none of these are focused on patients with acute PE. The PREFER in VTE disease registry (Prevention of Thromboembolic Events-European Registry in Venous Thromboembolism) was a prospective registry performed in seven European countries to assess the characteristics and the management of patients with venous thromboembolism, the use of health care resources, as well as the costs for 12 months treatment [31]. The study was not focused on patients with acute PE and not on the short-term management and course of the disease. As a consequence, limited data on acute phase management, mortality and decision making are available from this study. Similarly, the Global Anticoagulant Registry in the Field of Venous Thromboembolism Event (GARFIELD-VTE) is an international registry aimed at describing management and outcomes of patients with newly diagnosed deep vein thrombosis and/or PE up to 3 years from venous thromboembolism [32]. The primary objective of this registry is to determine the extent to which the treatment of venous thromboembolism varies in the real-world setting and to assess the impact of such variability on clinical and economic outcomes. The RIETE registry is an ongoing, prospective, dynamic cohort of consecutive patients presenting with symptomatic venous thromboembolism (deep-vein thrombosis, PE, or both) aimed at evaluating the clinical course of patients with acute venous thromboembolism treated with an anticoagulant. RIETE has no definite sample size or critical event committee and is mainly run at Angiology and Internal medicine Departments more than in the Cardiology or Emergency settings. ETNA-VTE is a single arm study on edoxaban treatment in routine clinical practice in patients with venous thromboembolism in Europe [33]. In this context, the focus on the acute phase management of patients with acute PE in a wide spectrum of clinical settings make COPE an original and novel study.

To enhance the scientific validity of our result, we planned to have a Critical Event Committee for central independent assessment of the cause of death based on a pre-defined charter. The Committee is unaware of physician classification and will receive all clinical documents related to the initial PE and to the circumstances of death. The purpose of central study outcome adjudication is to provide standardized, unbiased and blinded evaluation of investigator-reported endpoints, independently from investigator judgement [34]. Critical Event Committees are intended to enhance the scientific validity of a clinical trial through systematic, independent and standardized identification, processing and adjudication of study outcome events [35]. There are multiple lines of evidence indicating that central and independent adjudication of events may affect the results of a randomized trial. The presence of a CEC has been strongly advocated by regulatory authorities and requested in some instances for concern of bias in open-label studies [36, 37].

## Conclusion

Acute PE continues to be a leading cause of death and hospitalization all around the world despite continue advances in diagnostic and therapeutic strategies. In this context, contemporary data on in-hospital and 30-day mortality of patients with documented PE as well as information on guidelines adherence and its impact on clinical outcomes are needed to drive clinical research and inform health systems organization.

## Declarations

### Steering committee members

Cecilia Becattini, Michele Gulizia, Giancarlo Agnelli, Aldo P Maggioni, Francesco Dentali, Andrea Fabbri, Iolanda Enea, Fulvio Pomero, Maria Pia Ruggieri, Andrea di Lenarda.

### Critical event committee members

Maria Cristina Vedovati, M.D., Chair, Michele Duranti, M.D., Radiologist, Francesco Guercini, M.D., Paolo Groff, M.D., Melina Verso, M.D.
